# Dietary Mannoheptulose Increases Fasting Serum Glucagon Like Peptide-1 and Post-Prandial Serum Ghrelin Concentrations in Adult Beagle Dogs

**DOI:** 10.3390/ani5020365

**Published:** 2015-06-16

**Authors:** Leslie L. McKnight, Ryan Eyre, Margaret A. Gooding, Gary M. Davenport, Anna Kate Shoveller

**Affiliations:** 1 Centre for Nutrition Modelling, Department of Animal and Poultry Science, University of Guelph, Guelph, ON N1G 2W1, Canada; E-Mail: lmcknigh@uoguelph.ca; 2 The Iams Company, 6571 State Route 503 North, Lewisburg, OH 45338, USA; E-Mails: ryan.eyre@effem.com (R.E.); margaret.gooding@effem.com (M.A.G.); gary.m.davenport@gmail.com (G.M.D.)

**Keywords:** Beagle, body composition, dietary strategies, ghrelin, glucagon-like peptide-1, energy metabolism, indirect calorimetry, mannoheptulose, physical activity, satiety

## Abstract

**Simple Summary:**

There is increased interest in the use of nutraceuticals for weight management in companion animals. A nutraceutical can broadly be considered a food (or a part of) that provides a health benefit. Mannoheptulose (MH), a sugar found in avocados, is being investigated as a nutraceutical for dogs. In this study, dogs fed a diet containing MH had increased concentrations of blood biomarkers related to energy intake. In addition, dogs fed MH were less physically active than dogs fed a control diet. These findings suggest that dietary MH has the ability to alter energy intake and lower daily energy expenditure.

**Abstract:**

There is a growing interest in the use of nutraceuticals for weight management in companion animals. The purpose of this study was to determine the effects of mannoheptulose (MH), a sugar in avocados that inhibits glycolysis, on energy metabolism in adult Beagle dogs. The study was a double-blind, randomized controlled trial where dogs were allocated to a control (CON, n = 10, 10.1 ± 0.4 kg) or MH containing diet (168 mg/kg, n = 10, 10.3 ± 0.4 kg). Blood was collected after an overnight fast and 1 h post-feeding (week 12) to determine serum satiety related hormones and biochemistry. Resting and post-prandial energy expenditure and respiratory quotient were determined by indirect calorimetry (weeks 4 and 8). Physical activity was measured using an accelerometer (weeks 3, 7, 11). Body composition was assessed using dual X-ray absorptiometry (week 12). MH significantly (*p* < 0.05) increased fasting serum glucagon-like peptide-1 and post-prandial serum ghrelin. MH tended (*p* < 0.1) to increase fasting serum gastric inhibitory peptide and decrease physical activity. Together, these findings suggest that dietary MH has the ability to promote satiation and lowers daily energy expenditure.

## 1. Introduction

Companion animals are living increasingly longer lives, with over half the dogs in United States being over the age of six [1]. As in humans, advancing age is associated with metabolic diseases including obesity [2]. While the etiology of obesity is not fully understood, excessive energy intake and sedentary lifestyle are significant risk factors [3]. Canine obesity is commonly treated by nutritional management. Traditional management strategies are centered on total energy restriction (ER) and/or therapeutic (weight loss) diets. ER, without malnutrition, is the most robust and repeatable strategy for weight management across species [4]. Indeed, in a lifelong study of Labrador Retrievers, 25% ER decreased body weight and attenuated loss of lean body mass [5]. Furthermore, ER increased median and maximal lifespan and provided protection from age related disease development [6]. Despite these benefits, ER has limited success clinically due to the behavioural changes it elicits in pets (*i.e.*, begging, aggression).

In companion animals there is a growing interest in the use of nutraceuticals for weight management. While a universal definition does not exist, a nutraceutical can broadly be considered a food or a part of a food that provides a health benefit. Mannoheptulose (MH), a sugar in avocados that inhibits glycolysis, has been preliminarily investigated as a novel nutraceutical for dogs. Davenport *et al.* [7] observed lower fasting serum insulin in Labrador Retrievers fed a gelatin capsule containing 2, 10, and 20 mg/kg than dogs fed a placebo capsule or a capsule containing 1 mg/kg. In contrast, no changes in serum insulin or glucose or post-prandial energy expenditure (EE) were noted in Labrador Retrievers receiving a dietary dose of 2 mg/kg [8]. In Labrador Retrievers fed a dietary dose of 4 mg/kg, decreases in post-prandial energy expenditure and respiratory quotient (RQ) were observed [9]. Conversely, in Beagles fed an oral cocoa butter based supplement containing 8 mg/kg BW MH with a high carbohydrate relative to fat diet, an increase in post-prandial energy expenditure was noted [10]. Neither study observed changes in serum glucose or insulin with MH feeding. The mechanism by which MH would have differential effects on post-prandial energy expenditure is not fully understood. Furthermore, whether MH affects biomarkers of energy metabolism and satiety, aside from glucose and insulin, is unknown and may provide further information on the effect of MH on metabolism.

The primary objective of this study was to examine the effects of dietary MH (1.7 mg/kg) on outcomes related to energy metabolism in healthy adult Beagles. In addition, MH effects on serum biomarkers of whole body energy regulation were examined.

## 2. Experimental Section

### 2.1. Animals and Housing

All procedures were approved by the Institutional Animal Care and Use Committee of The Iams Company (Lewisburg, OH, USA). A total of 20 Beagles were used in this study. All dogs resided at The Iams Company (Lewisburg, OH, USA) and were considered healthy based on a general health evaluation by a licensed veterinarian prior to study. Dogs were pair housed in indoor runs (in the same building) with free access to water and indoor and outdoor runs. The indoor kennel was maintained on a 12 h light (06:30 to 18:30) and dark (18:30 to 06:30) cycle, in addition to natural light. Indoor temperature was set at 22 °C (range 18 °C to 24 °C) and humidity at 50% (range 40% to 70%) with 10 to 15 fresh air exchanges per hour. All indoor runs were equipped with raised canvas beds, toys and heated flooring. Outdoor runs were equipped with toys and play yard equipment. All dogs received 40 min of supervised group exercise and socialization in a separate fenced yard daily.

### 2.2. Study Design

The study was double-blind, randomized control trial, where adult, healthy dogs were randomly assigned to either a control (CON, 4 male, 6 female, 4.7 ± 0.3 year, body condition score 3 on a 5 point scale) or a mannoheptulose (MH, 5 male, 5 female, 4.1 ± 0.3 year, body condition score 3) containing diet. Groups were stratified by individual energy requirements established through historical feeding records. The study took place between May 2014 and September 2014 and included a 30 d dietary adaptation period and a 12 week study period. A summary of study measurements is presented in Table 1. The scheduling of study events reflects the need to accommodate multiple technical staff personnel on a single day. Furthermore, the indirect calorimetry method only allowed for 4 dogs to be measured per day. Therefore, the 20 dogs were divided into 5 groups of 4 dogs, with each diet represented on each day and staggered 1 d apart.

**Table 1:** Summary of study procedures.

Day of Study	Measurement or Procedure
Baseline	Fasting and post-prandial blood collection
	Dual X-ray absorptiometry to determine body composition
	Indirect calorimetry to determine energy expenditure and respiratory quotient
	Continuous physical activity monitoring using an accelerometer
Weeks 3, 7, 11	Continuous physical activity monitoring using an accelerometer
Weeks 4, 8	Indirect calorimetry to determine energy expenditure and respiratory quotient
Week 12	Fasting and post-prandial blood collection
Week 13	Dual X-ray absorptiometry to determine body composition

### 2.3. Diets and Feeding

Both diets, CON and MH, were made from identical ingredients and were similar in terms of nutrient content (Table 2). The MH diet was made by incorporating a water-soluble extract of flesh-only un-ripened fruit avocado (MH source) (Kemin Industries, Des Moines, IA, USA) into the CON diet to deliver a MH dose of approximately 170 mg/kg diet [11]. Few studies have administered MH orally to dogs and only one study has been conducted in Beagles. MH was given as an oral cocoa butter based supplement (8 mg MH/kg BW) to adult Beagles fed diets of different macronutrient compositions. Irrespective of diet, MH peaked 3 to 4 h after ingestion; however, an increase in post-prandial EE in Beagles fed a diet high in dietary carbohydrate relative to fat [10]. Animals in this study were fed a daily dietary dose of 5.8 mg MH/kg BW. This represents a cost effective dose that can be achieved in a commercial dry extruded pet food.

**Table 2:** Analyzed chemical profile (dry matter basis) of the control (CON) and mannoheptulose (MH) containing diets.

	CON	MH
Dry matter, %	91.7	91.9
Crude protein, %	27.2	27.5
Crude fat, %	15.9	15.6
Crude fiber, %	2.1	2.1
Ash, %	7.0	7.2
ME ^1^, kcal/g	3199	3219
Mannoheptulose, mg/kg	0	168

^1^ ME, metabolizable energy was calculated using modified Atwater factors, where protein, fat and carbohydrate provide 3.5, 8.5, and 3.5 kcal/g, respectively.

Animals were individually fed their daily ration in two meals (0700 h and 1300 h) and food intake was measured daily. Beginning 30 d prior to study initiation dogs were fed the CON diet and energy intakes were monitored daily and body weights weekly. To ensure all dogs received equivalent amounts of dietary energy (and MH), energy intakes were fixed at 125 kcal/(d·kg BW). This energy provision represents the median energy intake during the dietary adaptation period.

### 2.4. Body Composition Analysis

One week prior to study initiation and on week 13 of study, body composition analysis was completed using an DUAL x-ray densitometer (QDR4500, Hologic Inc., Bedford, MA, USA). Dogs were fasted overnight (18 h since last meal) and sedated using Dexmedetomidine (Dexdomitor, Pfizer, New York, NY, USA) at a dose of 0.02 mg/kg and Carprofen (Rimadyl, Pfizer, New York, NY, USA) at a dose of 2 to 4 mg/kg administered i.m. Propofol (Propoflo, Abbott, Abbott Park, IL, USA) at a dose of 5 to 7 mg/kg was administered i.v. for induction. Dogs were positioned on their sternum with the cranial aspect of ante brachium placed on the table to ensure the phalanges faced caudally. The hind limbs were extended with the tail placed straight and in between the hind limbs. A whole body scan was performed of the following regions: left arm, right arm, trunk, left leg, right leg and head. Scans were done in triplicate for each dog and the median value of the three scans was recorded. Following the scan, atipamezole (Antisedan^®^, Pfizer, New York, NY, USA) was administered to each dog at a dose of 0.2 mg/kg. Dogs were placed in a heated cage until fully recovered, once recovered, dogs were returned to their normal housing regime and monitored for 1 week for complications.

### 2.5. Serum Satiety Hormones and Biochemistry

A blood sample (3 mL) was drawn at baseline and on week 12 prior to the dog’s morning meal (fasting) for the measurement serum biochemistry and satiety related hormones. Another blood sample (3 mL) was drawn precisely 1 h after the meal (post-prandial) for measurement of satiety related hormones only. Three protease inhibitors Pefabloc (Sigma-Aldrich #76307, St. Louis, MO, USA), DPP IV Inhibitor (Millipore #DPP4, Billerica, MA, USA), and Protease Inhibitor Cocktail (Sigma-Aldrich #P2714, St. Louis, MO, USA) were prepared as per manufacturer’s instructions and immediately added to the blood to preserve satiety related gut hormones. The sample was mixed well, allowed to clot, and centrifuged (2700 rpm, 10 min) to separate serum. Five hundred microlitres of serum was divided into microcentrifuge tube aliquots and stored at −70 °C until analysis, avoiding any repeat freezing and thawing cycles.

Gut Hormones including ghrelin, gastric inhibitory polypeptide (GIP), glucagon like peptide-1 (GLP-1), glucagon, insulin, leptin, pancreatic peptide, and peptide YY were analyzed using commercially available MILLIPLEX Canine Gut Hormone Magnetic Bead Panel Assay Kit (#CGTMAG-98K, EMD Millipore, Billerica, MA, USA). All procedures were followed as per technical guidelines provided by the manufacturer and run on a Bio-Plex 200 instrument with Bio-Plex Manager software (Version 6.1, Bio-Rad, Hercules, CA, USA).

β-hydroxybutyrate was analyzed by enzyme assay using an Autokit 3-HB (Wako Diagnostics, Richmond, VA, USA) according to the manufacturer’s instructions. Samples were reacted and analyzed on the AU480 automated chemistry analyzer.

C-reactive protein was measured by double antibody ELISA. Samples (10 µL) were reacted with anti-CRP antibodies (1:50 dilution) adsorbed to the microtitre wells. After incubation, unbound protein was removed by washing and then an anti-CRP antibody conjugated with horseradish peroxidase was applied. The concentration of C-reactive protein in the sample was determined by measuring the absorbance at 450 nm. We used the Biotek Elx50 plate washer and the Biotek Synergy HT plate reader.

Total antioxidant capacity was measured using an antioxidant assay kit (Cayman Chemical Company #709001, Ann Arbor, MI, USA) according to manufacturer instructions. The assay measures the ability of antioxidants in the sample to inhibit the oxidation of the ability of serum antioxidants to inhibit the oxidation of 2,2′-azino-di-3-ethylbenzthiazoline sulphonate.

Serum biochemical measurements were measured using the Beckman Coulter AU480 Chemistry System (UV/vis spectrometry, Brea, CA, USA).

### 2.6. Indirect Calorimetry

Respiratory gas exchange measurements were conducted via whole-body indirect calorimetry at baseline and weeks 4 and 8 of study as described by McKnight *et al.* [10]. Prior to study initiation, dogs were acclimated over a 10-week period (1 to 8 h per week) to rest comfortably, without any apparent stress in the chamber with no excessive activity or movement. Prior to any measurements, the O_2_ and CO_2_ analyzers were calibrated with standard gases and dogs rested in the chamber for a minimum of 25 min to ensure adequate CO_2_ equilibration. Two fasting measurements were taken, after which dogs were fed their full daily ration of diet as a single meal (time 0) and gas exchange measurements continued for 14 h. Each chamber was sampled every 3 s over a 5 min period every 25 min. O_2_ and CO_2_ exchange and respiratory quotient (RQ) data were logged in real time using data acquisition software (Qubit Systems Inc., Kingston, ON, USA). Energy expenditure (EE) was calculated from O_2_ consumption and CO_2_ production (VO_2_ and VCO_2_) using the abbreviated Weir Equation [12].

### 2.7. Physical Activity Monitoring

Continuous, voluntary physical activity measurements were made using the Actical accelerometer (Philips Respironics, Bend, OR, USA) as described by McKnight *et al.* [9]. As only 10 Actical collars were purchased, 5 dogs from each dietary group (N = 10) were randomly selected to undergo activity monitoring. Measurements were taken for a 7-d period in order to capture weekday and weekend activity measurements. Sampling periods occurred one week prior to allocation to test diets (baseline) and on weeks 3, 7 and 11 of study. The average activity per minute was calculated during light (day, 06:30 to 18:30) and dark (night, 18:30 to 06:30) time periods.

### 2.8. Statistical Methods

All data were analyzed using SAS version 9.4 (SAS Institute, Cary, NC, USA) and reported as mean and pooled standard errors of the mean (SEM). Results were considered statistically significant if *p* < 0.05 and a statistical trend was defined as *p* value between 0.05 and 0.1.

A repeated measures ANCOVA model was used to test the main effects of study diet on weekly body weight. The diet-by-week interactions were included in the model and the baseline body weight, age at baseline and gender were included as a covariate. Each dog was considered a random effect yielding a block diagonal covariance matrix. Each covariates and its diet interaction was fitted in a separate model. The fit of each model was assessed and appropriate transformations and/or nonparametric analysis techniques were applied to the data, as necessary.

An ANCOVA model was used to test for main effects of study diet on serum biochemistry data, and physical activity. Baseline data were included as a covariate. Each dog was considered a random effect yielding a block diagonal covariance matrix.

Calorimetry data were fitted using the PROC MIXED procedure of SAS assuming fixed diet and time effects and dogs as random variables. Interactions between fixed effects were tested but only reported if significant.

## 3. Results and Discussion

### 3.1. Body Weight and Composition

All dogs maintained good general health. There was one incidence of diarrhea reported and one incidence of a foreign object in the stool documented. There were no differences in body weights or body composition between dietary groups at baseline or 13 weeks of study (Table 3). These results are not surprising as energy intakes were fixed (125 kcal/d·kg BW) throughout the duration of the study.

**Table 3:** Body weight and composition of adult Beagles at baseline and after 13 weeks of consuming a control diet (CON, n = 10) or a mannoheptulose containing diet (MH, 168 mg/kg, n = 10).

	CON	MH	SEM ^1^	*p*
Baseline				
Body weight, kg	10.1	10.3	0.4	0.64
Lean mass, kg	8.47	8.81	0.30	0.44
Fat mass, kg	1.55	1.51	0.21	0.91
Fat mass, %	15.3	14.1	1.8	0.64
Fat:lean	0.19	0.17	0.02	0.68
Week 13				
Body weight, kg	10.3	10.2	0.3	0.78
Lean mass, kg	8.56	8.40	0.15	0.44
Fat mass, kg	1.68	1.65	0.17	0.91
Fat mass, %	16.8	15.6	1.6	0.97
Fat:lean	0.20	0.20	1.2	1.0

^1^ Standard error of the mean.

### 3.2. Satiety Hormones and Serum Biochemistry

Fasting satiety hormones and serum biochemistry data are presented in Table 4. Fasting GLP-1 was significantly higher (*p* < 0.05) and GIP tended to be increased (*p* = 0.09) in dogs fed MH than those fed CON. There was no statistically significant effect of diet on any other fasting serum metabolites.

**Table 4:** Fasting serum satiety hormone and biochemistry in adult Beagles fed a control (CON, n = 10) or mannoheptulose containing diet (MH, 168 mg/kg, n = 10). ^1^

Metabolite	CON	MH	*p* Value
Insulin (pg/mL)	276.6 ± 51.8	262.8 ± 50.9	0.85
Glucagon (pg/mL)	110.2 ± 8.4	107.4 ± 7.9	0.80
Gastric inhibitory polypeptide (pg/mL)	1.13 ± 0.15	1.53 ± 0.19	0.09
Ghrelin (pg/mL)	172.7 ± 17.5	210.0 ± 41.3	0.41
Glucagon-like peptide-1 (pg/mL)	19.3 ± 2.7	33.0 ± 4.9	0.03
Leptin (pg/mL)	596 ± 89	642 ± 103	0.74
Pancreatic peptide (pg/mL)	32.9 ± 5.7	42.2 ± 9.6	0.43
Peptide YY (pg/mL)	200 ± 17	223 ± 10	0.24
C reactive protein (mg/L)	2.48 ± 1.7	1.95 ± 1.70	0.83
β-hydroxybutyrate (µmol/L)	50.84 ± 6.90	50.21 ± 12.05	0.96
Total Antioxidant Capacity (mmol/L)	1.16 ± 0.03	1.19 ± 0.03	0.47
Glucose (mmol/L)	5.35 ± 0.14	5.24 ± 0.17	0.63
Total Cholesterol (mg/dL)	194.82 ± 8.23	202.08 ± 6.33	0.50
Triglycerides (mg/dL)	52.76 ± 4.49	58.64 ± 6.60	0.46
Albumin/Globulin Ratio	1.25 ± 0.03	1.25 ± 0.02	0.85
Alkaline phosphatase (U/L)	39.05 ± 4.53	42.85 ± 3.84	0.52
Alanine Aminotransferase (U/L)	27.80 ± 3.24	33.30 ± 3.42	0.25
Aspartate Aminotransferase (U/L)	26.35 ± 2.14	27.15 ± 1.48	0.75
Albumin (g/dL)	3.16 ± 0.07	3.18 ± 0.06	0.83
BUN/Creatinine Ratio	16.49 ± 0.82	15.89 ± 0.65	0.58
Blood urea nitrogen (mg/dL)	12.09 ± 0.99	11.36 ± 0.75	0.55
Creatine kinase (U/L)	123.11 ± 24.85	92.99 ± 17.66	0.33
Creatinine (Mg/dL)	0.75 ± 0.08	0.70 ± 0.04	0.54
Gamma glutamyl transferase (U/L)	3.54 ± 0.18	4.00 ± 0.30	0.19
Lactate dehydrogenase (U/L)	54.19 ± 11.95	42.61 ± 9.17	0.45
Total bilirubin (mg/dL)	0.24 ± 0.03	0.25 ± 0.03	0.92
Total protein (G/dL)	5.68 ± 0.10	5.74 ± 0.09	0.64
Calcium (mg/dl)	9.71 ± 0.10	9.75 ± 0.09	0.76
Chloride (mEq/L)	114.07 ± 0.49	114.13 ± 0.63	0.94
Magnesium (mEq/L)	1.43 ± 0.05	1.52 ±0.04	0.15
Phosphorus (mg/dL)	3.12 ± 0.22	3.02 ± 0.22	0.75
Potassium (mEq/L)	4.25 ± 0.09	4.23 ± 0.07	0.85
Sodium (mEq/L)	146.76 ± 0.66	145.64 ± 0.56	0.20

^1^ Blood samples were taken at week 12 of study, data are mean ± SE.

Only satiety related hormones were measured post-prandial (1 h) (Table 5). Ghrelin was significantly greater in dogs fed MH and no other post prandial differences were observed. As expected, serum insulin, GLP-1, GIP, leptin, pancreatic peptide, and peptide YY concentrations increased post-feeding in both groups. These gut derived peptides collectively promote satiation in response to nutrient ingestion. Ghrelin, an appetite stimulating hormone released by the stomach, concentrations decreased post-feeding, which is in agreement with Bhatti *et al.* [13]. Glucagon concentration were unchanged from fasting concentrations, however, the ratio of insulin to glucagon was markedly increased post-feeding.

**Table 5:** Post-prandial (1 h post-feeding) serum satiety hormones in adult Beagles fed a control (CON, n = 10) or mannoheptulose containing diet (MH, 168 mg/kg, n = 10). ^1^

Metabolite	CON	MH	*p* Value
Insulin (pg/mL)	952.8 ± 145.3	785.9 ± 183.3	0.48
Glucagon (pg/mL)	124.4 ± 9.7	113.2 ± 9.3	0.41
Gastric inhibitory polypeptide (pg/mL)	35.4 ± 4.2	28.1 ± 4.5	0.24
Ghrelin (pg/mL)	73.0 ± 8.4	96.0 ± 6.7	0.04
Glucagon-like peptide-1 (pg/mL)	56.8 ± 4.8	62.4 ± 6.6	0.49
Leptin (pg/mL)	760 ± 113	722 ± 134	0.83
Pancreatic peptide (pg/mL)	125.7 ± 7.2	130.4 ± 9.8	0.66
Peptide YY (pg/mL)	260 ± 17	253 ± 12	0.71

^1^ Blood samples were taken at week 12 of study, data are mean ± SE.

To date, only dietary MH effects on blood glucose, insulin [7,8,9,10] and free fatty acids [9] have been examined. The present study is the first to investigate the effects of MH on other serum biomarkers of energy metabolism. Fasted dogs fed MH had significantly higher circulating GLP-1, and tended to have increased GIP concentrations. Both GLP-1 and GIP are released from intestinal epithelium in response to a meal and act as incretin hormones by potently amplifying insulin secretion while inhibiting glucagon secretion, even at low glucose concentrations [14]. However, MH did not affect fasting insulin or glucagon concentrations, the two primary targets of GLP-1. The inability of ingested MH to affect serum glucose or insulin concentrations is in agreement with McKnight *et al.* [8,9,10]. The mechanism by which MH may exert an effect on fasting metabolites is interesting, as MH effects on metabolism have been shown to be transient [9,15,16]. Indeed, MH completely disappears from plasma within 24 h of ingestion [7,10]. However, MH did not affect GLP-1 or GIP post-feeding (*i.e.*, when MH is present in circulation). Post-feeding, MH significantly increased circulating ghrelin concentrations only. Circulating ghrelin is increased prior to meal consumption and suppressed in response to meal ingestion [12,17]. However, the regulation and physiological significance of post-prandial ghrelin suppression is presently unclear [17]. One hypothesis may be that decreases in circulating ghrelin post-feeding may allow anorexigenic hormones such as GLP-1 to act more potently [18]. Therefore, the ability of MH to increase fasting GLP-1 and GIP and post-prandial ghrelin, may suggest MH promotes satiety and satiation. As energy intakes were fixed in this study to meet the maintenance energy requirements of dogs, the influence of MH on hunger, satiation and satiety could not be determined. Furthermore, a single blood sample was taken in this study 1 h post-feeding, yet MH concentrations have been shown to peak in plasma 2–4 h after ingestion [7,10]. Gooding *et al.* [19] reported an effect of MH on play motivation in cats which was hypothesized to be influenced by an underlying, central energy sensing system further substantiating a potential role of MH in hunger/satiety signaling. Future studies which examine the behaviours and neuronal responses associated with the consumption of MH in an unrestricted feeding paradigm and time course changes in serum biomarkers related to satiation, especially other orexigenic hormones, are warranted.

### 3.3. Indirect Calorimetry

There was no significant effect of diet on fasting or post-prandial EE or RQ on d 28 or d 56 of study (Table 6).

**Table 6:** Resting and post-prandial energy expenditure (EE) and respiratory quotient (RQ) on d 28 and 56 in adult Beagles fed either a control (CON, n = 10) or mannoheptulose containing diet (MH, 168 mg/kg, n = 10).

	CON	MH	SEM	*p* Diet	*p* Time
EE, kcal/(kg^0.75^·d)					
Resting, Week 4	87.0	86.0	4.1	0.88	-
Resting, Week 8	87.7	91.3	6.6	0.71	-
Post-prandial, Week 4	106.0	105.3	3.9	0.64	<0.01
Post-prandial, Week 8	103.0	99.0	4.5	0.53	<0.01
RQ					
Fasting, Week 4	0.72	0.70	0.001	0.22	-
Fasting, Week 8	0.71	0.69	0.01	0.22	-
Post-prandial, Week 4	0.81	0.80	0.01	0.35	<0.01
Post-prandial, Week 8	0.80	0.81	0.005	0.33	<0.01

^1^ Standard error of the mean.

Energy expenditure as measured by indirect calorimetry encompasses resting EE (REE), which represents the major component of daily EE (~70%) and is largely predicted by fat free mass [20], and a small amount of voluntary activity. When animals are well acclimated to rest calmly during calorimetry measurements, the thermic effect of feeding can also be measured. Indirect calorimetry does not include the energy expended during physical activity. Given the relatively short duration of study and that no changes in body weight or composition were noted over time, REE was not expected to be affected by diet. The REE values observed in this study are similar to those reported in Beagles [10,21].

Oral MH supplementation (8 mg/kg BW) has been shown to increase the thermic effect of feeding in adult Beagles fed a high carbohydrate relative to fat diet, but not when dogs consumed a low carbohydrate relative to fat diet [10]. In agreement with the present study, dietary MH (200 mg/kg of diet) did not affect EE post-feeding in adult Labrador Retrievers [8]. These results may suggest that the dietary dose of MH of ~2 mg/kg BW is insufficient to elicit changes in EE. Indeed, McKnight *et al.* [9] observed a transient decrease in post-prandial EE and RQ in Labrador Retrievers fed a dietary MH dose of 4 mg/kg BW. It is also likely that the dietary macronutrient composition and/or ingredients may impact the ability of dietary MH to induce changes in post-prandial EE.

### 3.4. Physical Activity Monitoring

Spontaneous physical activity data are presented in Table 7. Day time physical activity (activity per minute) tended to be lower in dogs fed MH (Week 11, *p* = 0.07, overall, *p* = 0.12) than those fed CON. Similarly, night time physical activity (activity per minute) also tended to be reduced in MH fed dogs (overall *p* = 0.08).

**Table 7:** Spontaneous physical activity in dogs in adult Beagles fed either a control (CON, n = 5) or mannoheptulose containing diet (MH, 168 mg/kg, n = 5).

	CON	MH	SEM ^1^	*p*
Day—Average activity per minute ^2^				
Week 3	245	207	31	0.41
Week 7	261	250	34	0.83
Week 11	276	189	34	0.07
Overall	260	214	20	0.12
Night—Average activity per minute				
Week 3	118	106	12	0.48
Week 7	162	133	14	0.14
Week 11	132	109	12	0.20
Overall	136	116	8	0.08

^1^ Standard error of the mean; ^2^ The average activity per minute was calculated during light (day, 06:30 to 18:30 h) and dark (night, 18:30 h to 06:30 h) time periods.

Physical activity, a variable component of daily EE, was measured using an accelerometer. Dogs in this study exhibited diurnal patterns of activity; specifically dogs were more active during the light than dark periods, irrespective of diet. This finding agrees with the activity patterns displayed by laboratory Beagles [22] and Labrador Retrievers [9]. MH tended to decrease day and night physical activity, particularly toward the end of study, week 11, opposed to week 3 or 7. Similarly, McKnight *et al.* [9] observed decreased daytime physical activity in Labrador Retrievers after six weeks of consuming a MH containing diet (400 mg/kg). These findings may suggest a relatively long metabolic and neural adaptation to MH feeding. It is important to note that this study measured locomotion only. Locomotion encompasses complex behaviours (*i.e.*, spontaneous activity, exploration and exercise) that are elicited by a wide range of internal and external stimuli. Therefore, a more critical analysis of physical activity and associated behaviours is necessary to fully understand the effects of MH.

## 4. Conclusions

In conclusion, dietary MH (168 mg/kg) increased fasting serum GLP-1, GIP and post-prandial ghrelin concentrations and tended to decrease spontaneous physical activity in healthy adult Beagles. Together these findings may suggest that dietary MH promotes satiety and lowers daily energy expenditure. However, energy intakes in this study were fixed to maintain dogs at an ideal body weight and condition. Future studies which examine the behaviours and neuronal responses associated with unrestricted energy intake (positive energy balance and weight gain), restricted energy intake (negative energy balance and weight loss), and in overweight or obese dogs are necessary to better understand the impact of dietary MH on whole body energy regulation. Outcomes of satiety hormones status, food intake and energy expenditure are of potential interest.

## References

[1] Case L.P., Daristotle L., Hayek M.G., Raasch M.F. (2001). Canine and Feline Nutrition.

[2] Lund E.M., Armstrong P.J., Kirk C.A., Klausner J.S. (2006). Prevalence and risk factors for obesity in adult dogs from private US veterinary practices. Int. J. Appl. Res. Vet. Med..

[3] Laflamme D.P. (2012). Obesity in dogs and cats: What is wrong with being fat?. J. Anim. Sci..

[4] Fontana L., Klein S. (2007). Aging, adiposity, and calorie restriction. JAMA.

[5] Kealy R.D., Lawler D.F., Ballam J.M., Mantz S.L., Biery D.N., Greeley E.H., Lust G., Segre M., Smith G.K., Stowe H.D. (2002). Effects of diet restriction on life span and age-related changes in dogs. JAVMA.

[6] Lawler D.F., Larson B.T., Ballam J.M., Smith G.K., Biery D.N., Evans R.H., Greeley E.H., Segre M., Stowe H.D., Kealy R.D. (2008). Diet restriction and ageing in the dog: major observations over two decades. Br. J. Nutr..

[7] Davenport G., Massimino S., Hayek M., Burr J., Ceddia M., Yeh C.-H., Roth G., Ingram D. (2010). Biological activity of avocado-derived mannoheptulose in dogs. FASEB J..

[8] McKnight L.L., Flickinger E.A., Davenport G.M., France J., Shoveller A.K. (2014). Dietary mannoheptulose has differential effects on fasting and post-prandial glucose oxidation in Labrador Retrievers. J. Appl. Anim. Res..

[9] McKnight L.L. (2014). The Effects of Oral Mannoheptulose Supplementation on Canine Energetics and Macronutrient Utilization. Ph.D. Thesis.

[10] McKnight L.L., Flickinger E.A., France J., Davenport G.M., Shoveller A.K. (2014). Mannoheptulose has differential effects on fasting and post-prandial energy expenditure and respiratory quotient in adult Beagle dogs fed diets of different macronutrient contents. J. Nutr. Sci..

[11] Massimino S.P., Niehoff R.L., Sarama R.J., Tribelhorn R.E. (2005). Processes for Preparing Plant Matter Extracts and Pet Food Compositions. US Patent.

[12] De Wier J.B. (1949). New methods for calculating metabolic rate with special reference to protein metabolism. J. Physiol..

[13] Bhatti S.F., Hofland L.J., van Koetsveld P.M., Van Ham L.M., Duchateau L., Mol J.A., van der Lely A.J., Kooistra H.S. (2006). Effects of food intake and food withholding on plasma ghrelin concentrations in healthy dogs. Am. J. Vet. Res..

[14] Holst J.J. (2007). The Physiology of Glucagon-like Peptide 1. Physiol. Rev..

[15] Issekutz B., Issekutz T., Elahi D. (1974). Effect of manno-heptulose on glucose kinetics in normal and gluco-corticoid treated dogs. Life Sci..

[16] Klain G.J., Meikle A.W. (1974). Mannoheptulose and fatty acid synthesis in the rat. J. Nutr..

[17] Briggs D.I., Andrews Z.B. (2011). Metabolic status regulates ghrelin function on energy homeostasis. Neuroendocrinology.

[18] Williams D.L., Cummings D.E. (2005). Regulation of ghrelin in physiologic and pathophysiologic states. J. Nutr..

[19] Gooding M.A., Davenport G.M., Duncan I.J.H., Atkinson J.L., Shoveller A.K. (2015). Dietary mannoheptulose (MH) enhanced play motivation after a 28 d feeding trial in cats. Appl. Anim. Behav. Sci..

[20] Johnstone A.M., Murison S.D., Duncan J.S., Rance K.A., Speakman J.R. (2005). Factors influencing variation in basal metabolic rate include fat-free mass, fat mass, age, and circulating thyroxine but not sex, circulating leptin, or triiodothyronine. Am. J. Clin. Nutr..

[21] Pouteau E.B., Mariot S.M., Martin L.J., Dumon H.J., Mabon F.J., Krempf M.A., Robins R.J., Darmaun D.H., Naulet N.A., Nguyen P.G. (2002). Rate of carbon dioxide production and energy expenditure in fed and food-deprived adult dogs determined by indirect calorimetry and isotopic methods. Am. J. Vet. Res..

[22] Siwak C.T., Tapp P.D., Zicker S.C., Murphey H.L., Muggenburg B.A., Head E., Cotman C.W., Milgram N.W. (2003). Locomotor activity rhythms in dogs vary with age and cognitive status. Behav. Neurosci..

